# Validated HPAEC-PAD Method for the Determination of Fully Deacetylated Chitooligosaccharides

**DOI:** 10.3390/ijms17101699

**Published:** 2016-10-10

**Authors:** Lidong Cao, Jinlong Wu, Xiuhuan Li, Li Zheng, Miaomiao Wu, Pingping Liu, Qiliang Huang

**Affiliations:** 1 Key Laboratory of Pesticide Chemistry and Application, Ministry of Agriculture, Institute of Plant Protection, Chinese Academy of Agricultural Sciences, No. 2 Yuanmingyuan West Road, Beijing 100193, China; caolidong@caas.cn (L.C.); lixiuhuan0822@163.com (X.L.); zhengli7seven@163.com (L.Z.); wumiaomiao2016@163.com (M.W.); 2 Institute for the Control of Agrochemicals, Ministry of Agriculture, No. 22 Maizidian Street, Beijing 110000, China; wjl_icama@126.com (J.W.); liupingping@agri.gov.cn (P.L.)

**Keywords:** chitooligosaccharides, HPAEC-PAD, method validation, quantification, hydrolysis

## Abstract

An efficient and sensitive analytical method based on high-performance anion exchange chromatography with pulsed amperometric detection (HPAEC-PAD) was established for the simultaneous separation and determination of glucosamine (GlcN)_1_ and chitooligosaccharides (COS) ranging from (GlcN)_2_ to (GlcN)_6_ without prior derivatization. Detection limits were 0.003 to 0.016 mg/L (corresponding to 0.4–0.6 pmol), and the linear range was 0.2 to 10 mg/L. The optimized analysis was carried out on a CarboPac-PA100 analytical column (4 × 250 mm) using isocratic elution with 0.2 M aqueous sodium hydroxide-water mixture (10:90, *v*/*v*) as the mobile phase at a 0.4 mL/min flow rate. Regression equations revealed a good linear relationship (*R*^2^ = 0.9979–0.9995, *n* = 7) within the test ranges. Quality parameters, including precision and accuracy, were fully validated and found to be satisfactory. The fully validated HPAEC-PAD method was readily applied for the quantification of (GlcN)_1–6_ in a commercial COS technical concentrate. The established method was also used to monitor the acid hydrolysis of a COS technical concentrate to ensure optimization of reaction conditions and minimization of (GlcN)_1_ degradation.

## 1. Introduction

Chitin is the second most abundant naturally occurring homopolysaccharide extracted from the exoskeleton of crustaceans and insects, and from fungal cell walls, etc. [1]. Chitosan, a linear heteropolysaccharide composed predominantly of β-1,4-linked-d-glucosamine (GlcN) and partially of β-1,4-linked *N*-acetyl-d-glucosamine (GlcNAc), is a deacetylation product of chitin. Chitosan is known to have attractive properties and has been used in many fields, such as agriculture, food, cosmetics, and medicine [2]. In recent decades, the study on chitosan has attracted interest in converting it into more soluble chitooligosaccharides (COS). These hydrolyzed oligomers of chitin and chitosan exhibit remarkable biological activities, such as antibacterial, antifungal, antioxidant, antitumor, and immunostimulant properties [3,4]. In addition, COS have been shown to induce various plant defense-related cellular responses and are therefore widely used in agriculture [4].

The biological activity of COS is known be highly structure-dependent. More specifically, the degree of polymerization (DP) and the degree of deacetylation (DD) influence the biological activity [4,5,6,7]. As a consequence, it has become imperative that analytical methods for determining COS quality, particularly the DP and single COS content, are developed. Qualitative techniques are essential. Electrospray ionization mass spectrometry and matrix-assisted laser desorption/ionization time-of-flight mass spectrometry (MALDI-TOF-MS) are frequently used for rapidly and reliably identifying COS with various DPs and DDs and for elucidating detailed structural information, such as molecular weight and average DP [7,8,9,10]. To separate and analyze a single COS, high-performance liquid chromatography (HPLC) coupled with various detectors was frequently used. The strong polarity and weak ultraviolet (UV) absorption of COS determine the choice of analytical column and detector. UV detector is commonly used with HPLC. Because of the presence of acetyl group, (GlcNAc)_n_ can be monitored using a UV detector by measuring the absorbance at around 210 nm with an amino column [11,12,13], a reversed-phase column [14], or a carbohydrate column [15,16]. To use UV detection with highly deacetylated COS, such as (GlcN)_n_, the absence of UV absorption groups in the chains necessitates further derivatization with a chromophore [17]. However, the derivatization process is time-consuming and may introduce experimental uncertainty.

As an alternative, a refractive index (RI) detector is preferentially adopted for HPLC analysis of COS, including (GlcN)_n_ and (GlcNAc)_n_. Amino columns [18,19,20,21,22,23,24] and size-exclusion columns [25,26] are typically coupled to this detector, which has low sensitivity and is not suitable for gradient elution. With these drawbacks, researchers are compelled to develop another methodology. Recently, hydrophilic interaction liquid chromatography (HILIC) was utilized as a powerful tool for separating carbohydrates [27,28]. Successful separation of COS with different DPs has been achieved under optimal elution conditions on HILIC columns, namely, amide and Click Maltose columns, coupled with an evaporative light-scattering detector (ELSD) [29,30,31,32]. In addition to the HPLC method, a capillary electrophoresis (CE) method using a laser-induced fluorescence detector has been developed for the separation of COS [33].

Although advances in analytical methods for COS have been achieved, detection by RI and ELSD have their inherent limitations, such as low detection sensitivity. Also, CE requires relatively expensive materials, and derivatization is time-consuming. High performance anion exchange chromatography (HPAEC) combined with pulsed amperometric detection (PAD) is a powerful, highly sensitive, and efficient system for separating underivatized carbohydrates [34]. Use of HPAEC-PAD for separating COS has previously been reported, but for the most part, they were used only for separating and analyzing (GlcNAc)_n_ [35,36,37,38]. Recently, van Munster reported a quantitative HPAEC-PAD method that allows fast separation of (GlcNAc)_1–6_ at the detection limits of 1–3 pmol and a linear range of 5–250 pmol [37]. Agger developed a fast and reliable quantitative HPAEC-PAD method for (GlcN)_1_, and the HPAEC elution patterns of (GlcNAc)_1–6_, and of (GlcN)_1,4–6_ were investigated [38]. Xiong [39] and Zhang [40] reported the separation and purity determination of (GlcN)_n_ using HPAEC-PAD. However, none of these HPAEC-PAD methods for assaying (GlcN)_1–6_ was validated and has found wide application.

In this study, a HPAEC-PAD method for separating and detecting underivatized (GlcN)_1–6_ was described. Parameters such as linearity, sensitivity, precision, and accuracy were fully validated. Moreover, this validated method was used to quantify (GlcN)_1–6_ in COS technical concentrate. The established method was also used to monitor acid hydrolysis of COS technical concentrate. Combined with MALDI-TOF-MS analysis, the method was used to qualitatively confirm the COS, even when the DP was greater than 6.

## 2. Results and Discussion

### 2.1. Optimization of Chromatographic Conditions

**Figure 1:**
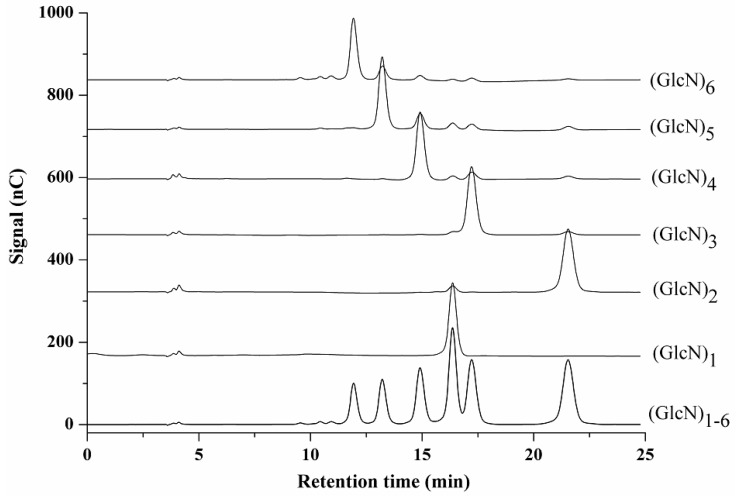
High-performance anion exchange chromatography with pulsed amperometric detection (HPAEC-PAD) chromatograms of (GlcN)_1–6_ standards.

When used with carbohydrates, HPAEC-PAD delivers high-resolution separation and sensitive detection. Native COS are undoubtedly suitable candidates for this method. Good separation depends primarily on the column, mobile phase composition, and flow rate. These three variables were systematically screened during optimization of chromatographic conditions, which was carried out using mixed (GlcN)_1–6_ standard solutions. Results showed that the CarboPac-PA100 afforded a better separation than CarboPac-PA10. Isocratic elution with 0.2 M aqueous sodium hydroxide–water mixture (10:90, *v*/*v*) at 0.4 mL/min provided satisfactory separation within 25 min. Using these parameters, symmetrical peaks were obtained for all standards. The COS with the highest DP eluted first, followed by COS with a lower DP, corresponding to results previously described for (GlcNAc)_1–6_ with the exception of (GlcN)_1_ [37,38] (Figure 1). These elution patterns differ from those obtained for normal homogenous oligosaccharides in ion chromatography, where retention times tend to increase with increasing DP. It is well known that in basic solution, carbohydrates behave as weak oxyanions, enabling them to be selectively eluted by HPAEC in a single run. The most important parameters influencing the retention are the number and position of hydroxyl groups, positional isomerism and the DP [34]. A hierarchy in the acidity of the various hydroxyl groups exists. For example, the hydroxyl on C1 (the reducing end) has the strongest effect on retention, while the second most important hydroxyl is at C2 [41]. In a COS, the hydroxyl at C2 is replaced by a primary amine or amide group, and these are neutral in alkaline conditions, thus not contributing to the ionic interactions with the quaternary-ammonium-bonded stationary phase [38]. As a consequence, the amount of negative charge per sugar monomer decreases as the DP increases. On the other hand, the ratio of the most acidic hydroxyl (at C1) to other hydroxyl groups in a given molecule decreases with increasing DP. Thus, the COS with higher DP will elute faster than those with lower DP except for (GlcN)_1_. These elution behaviors are similar to those seen with high-performance size exclusion chromatography (HPSEC), which has been widely used for polysaccharide separation. Separation using HPSEC relies on a differentiation in the time of permeation of the polysaccharides into and out of the pores of the column, and this is related to the molecular size [42]. Vårum reported that when separating COS on a size exclusion column, the one with the highest DP eluted first [43]. Even so, the separation principles of HPAEC and HPSEC are different. Separation of COS on amino columns depends primarily on hydrogen bond interactions between sugar compounds and NH_2_. Therefore, retention times normally increase with increasing DP [8,24,39], but this is different from the elution pattern using anion exchange.

It should be noted that the facile separation of (GlcN)_2–6_ is easily achieved. Much effort was spent determing the optimum condition for the separating (GlcN)_1_ from other COS. The elution order of (GlcN)_1_ varied with the changes in chromatographic conditions. In this study, (GlcN)_1_ eluted between (GlcN)_3_ and (GlcN)_4_. When using the CarboPac-PA10 column, the monomer peak either partially or completely overlapped with the (GlcN)_4_; using the CarboPac-PA100 column, it was closer to (GlcN)_3_. Recently, Agger reported using HPAEC-PAD to separate COS on theCarboPac-PA1 column, and the monomer peak either partially or completely overlapped with the (GlcN)_5_ [38]. On the CarboPac-PA1 column, (GlcNAc)_1_ eluted between (GlcNAc)_2_ and (GlcNAc)_3_, closer to the latter, when pure water was the mobile phase [37]. Using 5, 15 and 25 mM KOH solutions as the mobile phase, (GlcNAc)_1_ always eluted first and before (GlcNAc)_2–6_ [38].

### 2.2. Calibration and Method Validation

Quality parameters such as selectivity, sensitivity, linearity range, precision, and accuracy were fully studied. To investigate the selectivity, (GlcNAc)_1_, (GlcNAc)_2_ and a range of typical non-amino sugars, including glucose, mannose, fructose, and sucrose, were detected. The sugars can be separated from each component of (GlcN)_1–6_. However, if partly de-acetylated COS (PDACOS) are included, the present method faces challenges. While it is difficult to obtain all PDACOS authentic standards, it is also very labour-intensive to get a complete baseline separation between the components of (GlcN)_1–6_ and each of the PDACOS. Therefore, when assaying an actual COS sample, pre-analysis with MALDI-TOF-MS is suggested so that the general product profiles can be ascertained.

Linearity was tested by analyzing seven different concentrations of a single COS standard solution. The linear range for (GlcN)_1–6_ was approximately 0.2 to 10 mg/L, and all the calibration curves showed good linearity (*R*^2^ = 0.9979–0.9995, *n* = 7) in the tested range (Table 1). Slope coefficients, which are directly correlated to the detector response, progressively decreased with increasing DP. These response behaviors on a weight basis, were similar to what was found for inulooligosaccharides and maltosaccharides with DPs 3–7, reported by Borromei [44] and Koch [45]. This could be reasonably explained by the decrease in molarity of the repeated unit with increasing DP at the same mass concentration. When the molar response (peak area/pmol) was evaluated, the slope coefficients of the components of (GlcN)_1–6_ were 0.1035, 0.1032, 0.1399, 0.1441, 0.1482, and 0.1240, in order of DP, exhibiting a smaller difference compared to a mass detector response. The limits of detection (LOD) and quantification (LOQ) were defined as the minimum amounts at which the analyte can be reliably detected and quantified. Typical signal-to-noise (S/N) ratios of the LOD and LOQ were 3 and 10, respectively. Diluted solutions (low concentrations) of the (GlcN)_1–6_ were injected to determine S/N ratio. Then the LOD and LOQ were calculated. The LOD and LOQ ranged from 0.003 to 0.016 (corresponding to 0.4–0.6 pmol) and 0.009 to 0.054 mg/L (corresponding to 1.2–2.0 pmol), respectively.

Precision was determined by measuring known amounts of the mixed (GlcN)_1–6_ standards and real samples of COS technical concentrate. To establish repeatability (intraday) and intermediate (interday) precision for the mixed (GlcN)_1–6_ standards, variations in terms of peak areas and retention times at three concentration levels were determined (Table 2). In HPAEC, critical influence factors for retention and signal response are the state of the column, mobile phase, and gold electrode. To obtain repeatability and intermediate precision, it is crucial that elution and detector response conditions be held constant. Repeatability was assessed using seven replicates in one HPAEC run. Under repeatability conditions, retention times and integrated peak areas of all tested analytes were stable with 0.1–0.6 and 0.9–4.7%RSD, respectively. Intermediate precision was assessed from nine determinations (three determinations daily over three days) using the same equipment, but performed by two individuals on three consecutive days using three separately prepared batches of mobile phase. Under intermediate precision conditions, retention times and integrated peak areas of all tested analytes were stable with 0.4–1.0 and 1.7–6.6%RSD, respectively. These are slightly higher than what was found for repeatability. Method precision was also evaluated by comparing the variations among seven replicate determinations of the same batch of COS technical concentrate with the Horwitz value (%*RSDr*) [46,47,48,49]. All the %RSD values of (GlcN)_1–6_ determinations were less than the corresponding %*RSDr* (Table 3), demonstrating that the established method is precise. Method accuracy was determined using the standard addition method under optimized conditions, and it was found to be satisfactory, with the recoveries ranging from 91.2% to 103.9% (Table 4). These validation results indicate that this HPAEC-PAD method is precise, accurate and sensitive for the simultaneous quantitative determination of COS, at least (GlcN)_1–6_.

### 2.3. Method Application

**Figure 2:**
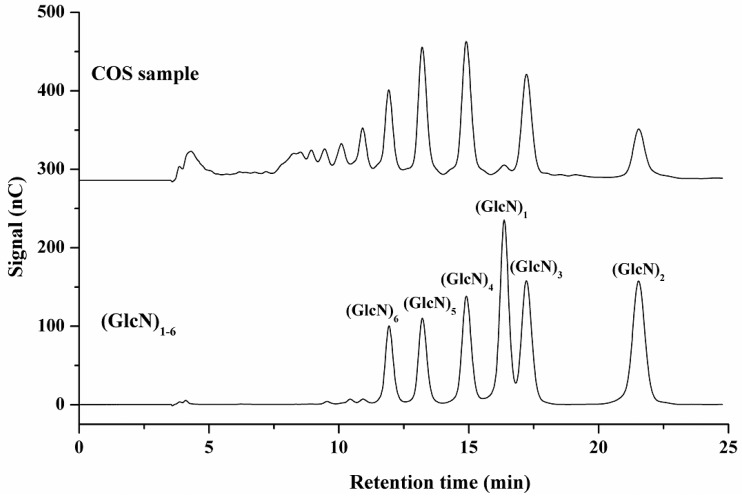
HPAEC-PAD chromatograms of COS technical concentrate and (GlcN)_1–6_ standards.

This HPAEC-PAD method for unlabeled COS separation can provide relatively quick pre-analysis of commercial and lab-synthesized COS. The distribution profile of COS and general DP can be facilely achieved with the help of COS standards and use of MALDI-TOF-MS. Quantification of a single COS in a commercial COS technical concentrate can be efficiently determined using this HPAEC-PAD method. Representative chromatograms of COS technical concentrate and (GlcN)_1–6_ mixed standards are shown in Figure 2. The COS with different DP were well separated, and the qualitative and quantitative determinations were feasible. Based on the elution behavior (highest DP eluting first) and the MALDI-TOF-MS results, the two adjacent peaks in front of (GlcN)_6_ can be identified as (GlcN)_7_ and (GlcN)_8_. Detailed results of the assay of (GlcN)_1–6_ with seven replicates are summarized in Table 3.

**Figure 3:**
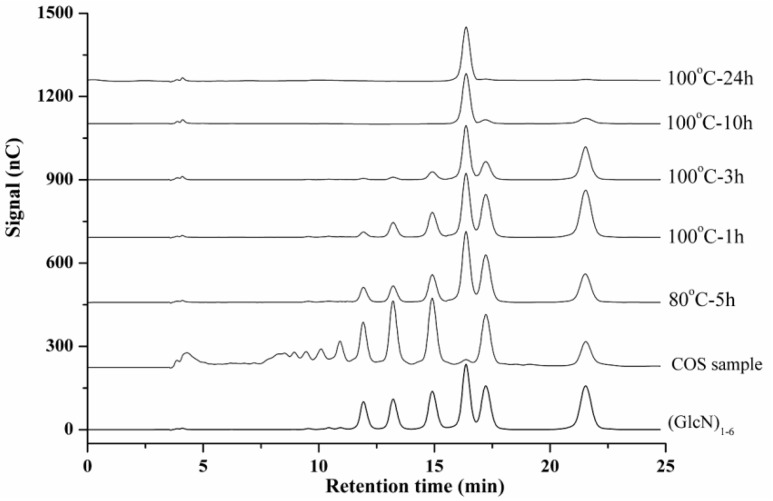
HPAEC-PAD chromatograms of (GlcN)_1–6_ standards, COS technical concentrate, and the acid hydrolysis of COS technical concentrate under various conditions.

Because of the unavailability of some single COS standards, quantification of chitosan or COS must always adopt an indirect method, namely, whereby chitosan or COS are hydrolyzed into glucosamine followed by the subsequent characterization of the monomer. Acid hydrolysis using concentrated hydrochloride (HCl) is the most widely used method, because it is effective in both depolymerization and deacetylation of chitosan or COS [50]. However, the degradation of (GlcN)_1_ during acid hydrolysis will result in the decrease of recovery of chitosan or COS. Therefore, this hydrolysis procedure should be efficiently monitored and carefully optimized so that chitosan or COS are completely hydrolyzed with minimun degradation of (GlcN)_1_. This HPAEC-PAD method is undoubtedly suitable for monitoring the hydrolysis procedure. To demonstrate its applicability, the method was used to trace the acid hydrolysis of COS technical concentrate. Typical HPAEC-PAD chromatograms of the reaction mixtures under various conditions are shown in Figure 3. It can be seen that COS hydrolysis is greatly influenced by temperature. At 80 °C, hydrolysis proceeded slowly; after 5 h, COS with higher DPs (4–6) remained. However, at 100 °C, depolymerization proceeded quickly, and COS with higher DPs were hydrolyzed faster than those with lower DPs. After 10 h, only trace amounts of (GlcN)_2_ and (GlcN)_3_ remained. After 24 h, all COS were completely hydrolyzed into (GlcN)_1_. These results demonstrate that this method offers a quick and efficient means to monitor hydrolysis of COS. Research into detailed hydrolysis kinetics and screening for optimal conditions are in progress.

### 2.4. MALDI-TOF-MS Analysis

**Figure 4:**
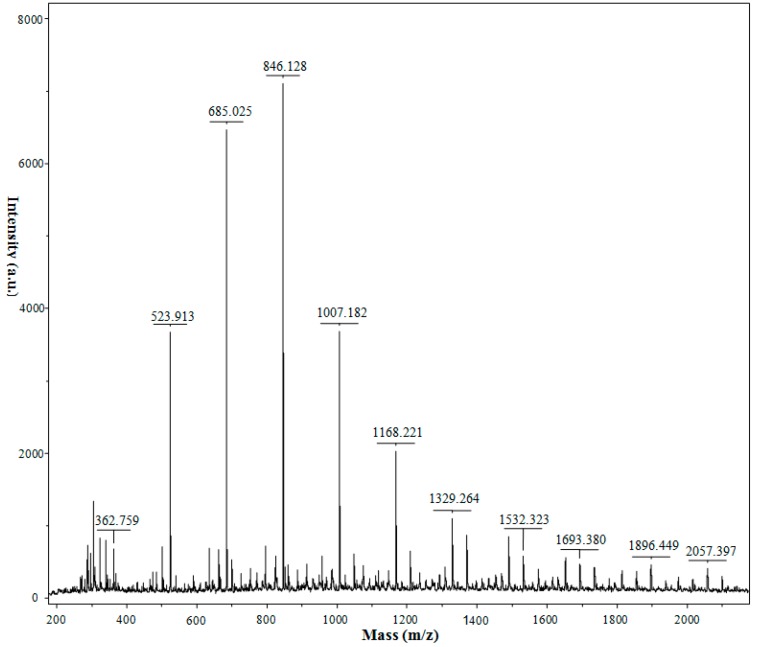
The matrix-assisted laser desorption/ionization time-of-flight mass spectrometry (MALDI-TOF-MS) of chitooligosaccharides technical concentrate.

Though HPAEC-PAD offers little in the way of direct structural identification, it is capable of detecting both neutral and charged oligosaccharides, making MALDI-TOF-MS use quite complementary [51]. MALDI-TOF-MS, a process of soft-ionization, causes little or no fragmentation of analyte, allowing the molecular ions of analyte to be identified, even within mixtures. In this study, MALDI-TOF-MS was employed to qualitatively determine the COS technical concentrate, revealing a tentative structural identification (Figure 4). All spectra exhibited monomodal mass distributions without fragmentation. With the molecular weight of the repeated units of glucosamine (GlcN) and N-acetylglucosamine (GlcNAc), the DP and molecular composition of each COS detected can be obtained and predicted (Table 5). Based on the MALDI-TOF-MS results, the two adjacent peaks in front of (GlcN)_6_ can be identified as (GlcN)_7_ and(GlcN)_8_. The peaks in front of (GlcN)_8_ are mainly partially deacetylated COS with the DPs higher than 8.

## 3. Experimental Section

### 3.1. Materials

Five COS standards in their hydrochlorides, (GlcN)_2_ (≥98%), (GlcN)_3_ (≥96%), (GlcN)_4_ (≥92%), (GlcN)_5_ (≥90%), and (GlcN)_6_ (≥80%), were obtained from Dalian Glycobio Co., Ltd. (Dalian, China). Glucosamine hydrochloride (≥98%) was purchased from National Institutes for Food and Drug Control (Beijing, China). COS technical concentrate was provided by the National Quality Supervision and Inspection Center of Pesticides (Beijing, China). Ultra-pure water was prepared using a MilliQ-50 SP reagent water system (Millipore Corporation, Bedford, MA, USA). Sodium hydroxide solution (50%, *w*/*w*) was purchased from Alfa Aesar (Tianjin, China) Co., Ltd. (Tianjin, China). Primary stock solution of each single COS standard was prepared by dissolving the COS in water at a concentration of 200 mg/L. All stock solutions were stored in the refrigerator at 4 °C until required for use. Working standard solutions were prepared as needed by appropriately diluting concentrated stock solutions with water. The COS technical concentrate was accurately weighed and dissolved in water to prepare stock sample solution. The working sample solutions were prepared by dilution with water.

### 3.2. HPAEC-PAD Analysis

An ICS-3000 system (Dionex, Sunnyvale, CA, USA) equipped with a CarboPac-PA100 guard column (4 × 50 mm) and a CarboPac-PA100 analytical column (4 × 250 mm) was used for HPAEC-PAD. The columns were washed for at least 20 min at 1.0 mL/min using 0.2 M NaOH when no sample was injected. Column equilibration was performed by maintaining starting conditions for at least 20 min before sample injection. Detection was accomplished using a PAD with a gold working electrode and an Ag/AgCl reference electrode. A standard carbohydrate quadruple potential waveform was used. The gold electrode was regularly maintained. Integration was performed using a Chromeleon 6.8 chromatography data system.

Isocratic elution was optimized by employing water (eluent A) and 0.2 M aqueous sodium hydroxide (eluent B). To prepare eluent B, approximately 1800 mL of water was added to a 2 L plastic bottle, then 20.9 mL of 50% (*w*/*w*) NaOH solution was pipetted from the middle of the stock solution into the water. This mixture was diluted up to the 2 L line, then the bottle was gently rotated to mix the eluent in a short period of time. All eluents were degassed and pressurized with high-purity nitrogen to prevent dissolution of carbon dioxide and subsequent production of carbonate, which would act as a displacing ion and shorten retention times. Furthermore, the eluent could not be used after one week. Additional eluent was meticulously prepared the same way each time to ensure consistency. Elution was carried out at a flow rate of 0.4 mL/min and 25 μL was injected. The concentration of each COS in the samples was calculated using a calibration curve that gave the relationship between the amount of analyte and the peak area. All analyses were carried out in duplicate.

### 3.3. Calibration

To assess linearity, calibration curves were plotted by partial least squares method on the analytical data of peak area and concentration, using analyte standards (*n* = 7) covering the concentration range of 0.2–11.5 mg/L for (GlcN)_1_, 0.2–9.8 mg/L for (GlcN)_2_, 0.2–10.1 mg/L for (GlcN)_3_, 0.2–10.6 mg/L for (GlcN)_4_, 0.2–10.6 mg/L for (GlcN)_5_ and 0.2–9.9 mg/L for (GlcN)_6_. The dilute standard solution was further diluted to the known low concentration with water for signal-to-noise (S/N) ratio determination. The limits of detection (LOD) and quantification (LOQ) were determined as the minimum concentrations resulting in signal-to-noise (S/N) ratios of 3 and 10, respectively.

### 3.4. Method Validation

Selectivity was assessed by determining chromatographic retention times of (GlcN)_1–6_, (GlcNAc)_1–2_, and a range of typical non-amino sugars, including glucose, mannose, and fructose.

Precision was determined by measuring known amounts of the mixed (GlcN)_1–6_ standards and a real sample of the COS technical concentrate as repeatability (intraday) and intermediate (interday) precision. For the mixed (GlcN)_1–6_ standards solution, the precision of the proposed method in terms of retention time and peak area was determined. Repeatability was assessed using seven replicates in one HPAEC run. Intermediate precision was evaluated from nine determinations (three determinations daily over three days) using the same equipment, but performed by two individuals on three consecutive days using three separately prepared batches of mobile phase. Variations were expressed using relative standard deviation (%RSD). Both repeatability and intermediate precision were determined at three concentrations levels of the mixed (GlcN)_1–6_ standards solution. To prepare three concentrations levels, 0.4 mL of each COS standard stock solution was pipetted into 25, 50 and 100 mL volumetric flasks, respectively, and the standardized volume was made up with water. For the real sample COS technical concentrate, the contents of (GlcN)_1–6_ were determined under the prescribed conditions. The coefficient of variations of seven replicate determinations of the same batch of COS technical concentrate are compared with the Horwitz value (%*RSDr*) [46]. The Horwitz equations are described as follows:

(1) $$\%RSD_{R}=2^{\left(1-0.5\log_{10}C\right)}$$

(2) $$\%RSDr=\%RSD_{R}\times 0.67$$

where %*RSD_R_* represents the inter-laboratory coefficient of variation (CV), %*RSDr* represents the repeatability CV, and *C* represents the concentration of the analyte in the sample as a decimal fraction.

The recovery test, determined by the standard addition method, was used to evaluate method accuracy. A known amount of individual COS working standard solution was added to a predetermined amount of the COS technical concentrate, and the spiked sample was assayed. The total amount of each analyte was calculated from the corresponding calibration curve, and recovery was calculated using the formula: recovery (%) = (observed amount − original amount)/spiked amount × 100%. Solutions were prepared by accurately weighing 20 mg of the test item into three 100 mL volumetric flasks spiked with 1.0, 2.0 and 3.0 mL of the individual COS stock standard solution, respectively, and the standardized volume was made up with water. From each, 1.0 mL was volumetrically transferred to a 10 mL volumetric flask, and the standardized volume was made up with water. Three determinations were performed for each standard addition. Each determination was injected in duplicate.

### 3.5. Hydrolysis of COS

Hydrolysis was performed on 30 mg COS technical concentrate, originally in powdered form. An accurately weighed portion was transferred to a 25 mL thick-walled glass tube (Synthware, Beijing, China), 3 mL of 6 N hydrochloride solution was added, and the mixture was flushed with nitrogen to remove oxygen in order to prevent oxidation reactions. The tube was sealed with a threaded Teflon stopper. Hydrolysis was performed at the setting temperature (80 or 100 °C) to generate (GlcN)_1_. The reaction procedure was monitored using HPAEC-PAD.

### 3.6. MALDI-TOF-MS Analysis

For qualitative confirmation of COS, MALDI-TOF-MS (Autoflex III TOF MS, Bruker Corporation, Billerica, MA, USA) was used in the positive mode using reflectron optics. 2,5-Dihydroxybenzoic acid was used as a matrix, and the COS were detected as singly charged sodium adducts.

## 4. Conclusions

A specific and sensitive analytical method for detecting and quantifying COS is essential for evaluating their structure-function relationship and potential applications in both glycobiology and enzymology. In this work, an efficient, sensitive, and quick HPAEC-PAD method was established and demonstrated as suitable for separating, identifying, and quantifying (GlcN)_1–6_ without derivatization within 25 min. High sensitivity, satisfactory linearity, precision and accuracy were achieved. The proposed method was readily applied for quantitative determination of (GlcN)_1–6_, providing a useful method for routine analysis of COS for quality control and biological research purposes. This method can also be used for monitoring COS hydrolysis, which is essential for optimizing hydrolysis conditions to minimize the degradation of (GlcN)_1_. However, this method cannot address the issue of having mixed partially deacetylated COS because of co-elution. The method is applicable only to fully deacetylated COS, or COS with rather high DD if determination errors can be acceptable.

## Figures and Tables

**Table 1:** Linearity of the calibration curve of (GlcN)_1–6_ standards.

Compound	Linear Range (mg/L)	Calibration Curve ^a^	*R*^2^	LOD (mg/L)	LOQ (mg/L)
(GlcN)_1_	0.2–11.5	*y* = 14.455*x* + 0.5347	0.9994	0.003	0.009
(GlcN)_2_	0.2–9.8	*y* = 7.6363*x* + 1.0974	0.9984	0.008	0.027
(GlcN)_3_	0.2–10.1	*y* = 7.0084*x* + 0.9652	0.9988	0.008	0.027
(GlcN)_4_	0.2–10.6	*y* = 5.4722*x* + 0.6928	0.9979	0.011	0.035
(GlcN)_5_	0.2–10.6	*y* = 4.5079*x* + 0.0593	0.9995	0.013	0.045
(GlcN)_6_	0.2–9.9	*y* = 3.1615*x* + 0.3702	0.9992	0.016	0.054

^a^ y and x refer to the signal response (nC) and mass concentration (mg/L), respectively. LOD: limit of detection; LOQ: limit of quantification.

**Table 2:** Determination of method precision under repeatability (intraday) and intermediate precision (interday) conditions given as RSD (%) of peak area and retention time.

Analyte	Repeatability (*n* = 7)						Intermediate Precision (*n* = 9)					
	Peak Area			Retention Time			Peak Area			Retention Time		
	C1	C2	C3	C1	C2	C3	C1	C2	C3	C1	C2	C3
(GlcN)_1_	2.77	2.71	2.06	0.11	0.40	0.28	2.51	2.67	2.29	0.41	0.59	0.67
(GlcN)_2_	4.09	2.38	4.16	0.12	0.53	0.34	4.17	3.63	4.27	0.54	0.54	0.90
(GlcN)_3_	3.16	2.22	2.91	0.14	0.54	0.35	2.92	2.79	3.17	0.59	0.58	0.92
(GlcN)_4_	2.66	0.99	2.95	0.11	0.57	0.36	2.38	2.65	2.66	0.58	0.58	0.97
(GlcN)_5_	2.96	0.90	3.24	0.13	0.58	0.38	2.95	2.66	4.04	0.60	0.60	0.99
(GlcN)_6_	4.67	1.54	3.95	0.12	0.56	0.40	4.71	1.74	6.63	0.66	0.66	1.02

RSD: relative standard deviation. C1 (mg/L): (GlcN)_1_ (3.2); (GlcN)_2_ (3.1); (GlcN)_3_ (3.2); (GlcN)_4_ (3.1); (GlcN)_5_ (3.2); (GlcN)_6_ (3.3). C2 (mg/L): (GlcN)_1_ (1.6); (GlcN)_2_ (1.6); (GlcN)_3_ (1.6); (GlcN)_4_ (1.6); (GlcN)_5_ (1.6); (GlcN)_6_ (1.7). C3 (mg/L): (GlcN)_1_ (0.8); (GlcN)_2_ (0.8); (GlcN)_3_ (0.8); (GlcN)_4_ (0.8); (GlcN)_5_ (0.8); (GlcN)_6_ (0.8).

**Table 3:** Determination of each chitooligosaccharides (COS) in COS technical concentrate containing (GlcN)_1–6_.

Analyte	Content (%, m/m) ^a^							Mean (%)	%RSD	%*RSDr*
	1	2	3	4	5	6	7			
(GlcN)_1_	0.16	0.16	0.17	0.16	0.16	0.15	0.16	0.16	3.19	3.53
(GlcN)_2_	2.52	2.44	2.52	2.59	2.53	2.55	2.50	2.52	1.83	2.33
(GlcN)_3_	5.44	5.55	5.66	5.55	5.56	5.64	5.74	5.59	1.70	2.07
(GlcN)_4_	9.73	9.86	9.55	9.67	10.09	9.78	9.67	9.76	1.80	1.90
(GlcN)_5_	11.42	11.08	11.21	11.26	10.93	11.07	10.92	11.13	1.64	1.86
(GlcN)_6_	8.36	8.47	8.54	8.25	8.37	8.48	8.62	8.44	1.49	1.94

^a^ Content (%, *m*/*m*): Mass percentage of the individual COS in COS technical concentrate.

**Table 4:** Method accuracy for determining each COS in COS technical concentrate containing (GlcN)_1–6_.

Analyte	Recovery (%)		
	Spiked C1	Spiked C2	Spiked C3
(GlcN)_1_	91.16 ± 3.22	103.74 ± 0.79	95.47 ± 2.73
(GlcN)_2_	97.59 ± 0.96	97.81 ± 2.93	95.98 ± 1.53
(GlcN)_3_	94.01 ± 1.08	95.93 ± 0.13	99.67 ± 3.26
(GlcN)_4_	95.08 ± 0.60	98.97 ± 2.21	98.42 ± 2.35
(GlcN)_5_	94.94 ± 0.63	97.94 ± 3.02	103.94 ± 2.16
(GlcN)_6_	97.11 ± 0.54	94.17 ± 0.45	97.42 ± 2.46

All values were given as mean recovery (*n* = 3) ± SD. SD: Standard deviation; C1: 0.2 (mg/L); C2: 0.4 (mg/L); C3: 0.6 (mg/L).

**Table 5:** MALDI-TOF-MS data for COS technical concentrate.

Chitooligosaccharides	*m*/*z* [M + Na]^+^	DP
(GlcN)_2_	363	2
(GlcN)_3_	524	3
(GlcN)_4_	685	4
(GlcN)_5_	846	5
(GlcN)_6_	1007	6
(GlcN)_5_(GlcNAc)_1_	1049	6
(GlcN)_7_	1168	7
(GlcN)_6_(GlcNAc)_1_	1210	7
(GlcN)_8_	1329	8
(GlcN)_7_(GlcNAc)_1_	1371	8
(GlcN)_9_	1490	9
(GlcN)_8_(GlcNAc)_1_	1532	9
(GlcN)_7_(GlcNAc)_2_	1574	9
(GlcN)_10_	1651	10
(GlcN)_9_(GlcNAc)_1_	1693	10
(GlcN)_8_(GlcNAc)_2_	1735	10
(GlcN)_7_(GlcNAc)_3_	1777	10
(GlcN)_11_	1812	11
(GlcN)_10_(GlcNAc)_1_	1854	11
(GlcN)_9_(GlcNAc)_2_	1896	11
(GlcN)_8_(GlcNAc)_3_	1938	11
(GlcN)_12_	1973	12
(GlcN)_11_(GlcNAc)_1_	2015	12
(GlcN)_10_(GlcNAc)_2_	2057	12
(GlcN)_9_(GlcNAc)_3_	2099	12

DP: Degree of polymerization; MALDI-TOF-MS: Matrix-assisted laser desorption/ionization time-of-flight mass spectrometry.
